# Quality of life of therapies for hormone receptor positive advanced/metastatic breast cancer: Regulatory aspects and clinical impact in Europe

**DOI:** 10.1016/j.breast.2021.07.008

**Published:** 2021-07-12

**Authors:** L. Moscetti, I. Sperduti, A. Frassoldati, A. Musolino, C. Nasso, A. Toss, C. Omarini, M. Dominici, F. Piacentini

**Affiliations:** a Department of Oncology-Hematology, University Hospital of Modena, Modena, Italy; b Department of Bio-Statistics, RCCS Regina Elena National Cancer Institute, Italy; c Department of Oncology, Ospedale Sant'Anna di Cona, Ferrara, Italy; d Medical Oncology and Breast Unit, University Hospital of Parma, Department of Medicine and Surgery, University of Parma, Parma, Italy; e Division of Medical Oncology, Department of Medical and Surgical Sciences for Children & Adults, University Hospital of Modena, Modena, Italy; f Gruppo Oncologico Italiano per la Ricerca Clinica (GOIRC), Italy

## Abstract

In recent years, the number of trials incorporating health-related quality of life (HRQoL) data has increased. The impact of HRQoL on regulatory decision making in the European context and on clinical practice is not well established. We conducted an analysis of the role of QoL data extracted from the clinical trials of the drugs approved for hormone receptor positive/HER2-negative advanced/metastatic breast cancer (mBC). The results from the HRQoL were collected and a meta-analysis was performed to evaluate the impact of experimental drugs compared to standard treatments. The results showed a non-detrimental effect in HRQoL from the new treatments. As regards the approval process, from an examination of the European Medicine Agency (EMA) documents, HRQoL was reported nonextensively and contained and discussed in the European assessment reports (EPARs) for eleven trials in the approval process and cited in three cases in the EPARs and summary of medicinal product characteristics (SmPC). An effort should be made by all the stakeholders to increase the visibility of the HRQoL results in order to allow increased consideration in the approval process to make QoL data more easily and visibly available for the clinician and the patients. The evaluation should be reflected in the SmPC in order to increase the amount of information provided to the physician.

## Highlights

• The impact of HRQoL on regulatory decision making in Europe is not well established.
• PROs allow the patient's perspective to be incorporated in assessing benefits/harms of treatments.
• HRQoL data are contained/discussed not extensively in EPARs and SmPCs.
• The meta-analysis on ten trials showed an overall improvement in global QoL.
• An effort should be made to make QoL data more easily and visibly available.

## 1 Introduction

Patient-reported outcomes (PRO) allow the patient's perspective to be incorporated in clinical trials to assess benefits and harms of treatments, informing regulatory and clinical decisions [1,2]. PROs are often collected in major recent drug developments but their impact on the approval process and their influence in clinical practice are not well known. Indeed, regulatory decisions are usually based on clinical outcomes such as progression free survival (PFS) or overall survival (OS), which are still the primary endpoints of most trials. Health-related quality of life (HRQoL) is a PRO that has habitually been included as a secondary endpoint in clinical trials. There are a number of questionnaires and tools used to collect PROs that are generally well established and include generic as well as disease-specific modules. However, the variety of tools available adds to the complexity of contextualizing results across trials. In recent years, the number of trials incorporating HRQoL data has increased, also driven by the need for such data in health-economic evaluations [3]. The aim of this paper is to describe how the HRQoL data submitted to the European Medicine Agency (EMA) for the initial marketing authorization (MA) for the treatment of hormone receptor positive/HER2-negative (HR+/HER2-) advanced/metastatic breast cancer (mBC), are considered in the approval process of the European regulatory context, and to conduct a meta-analysis to evaluate the impact on HRQoL of brand new treatments compared to the standard treatments.

## 2 Methods

**Fig. 1:**
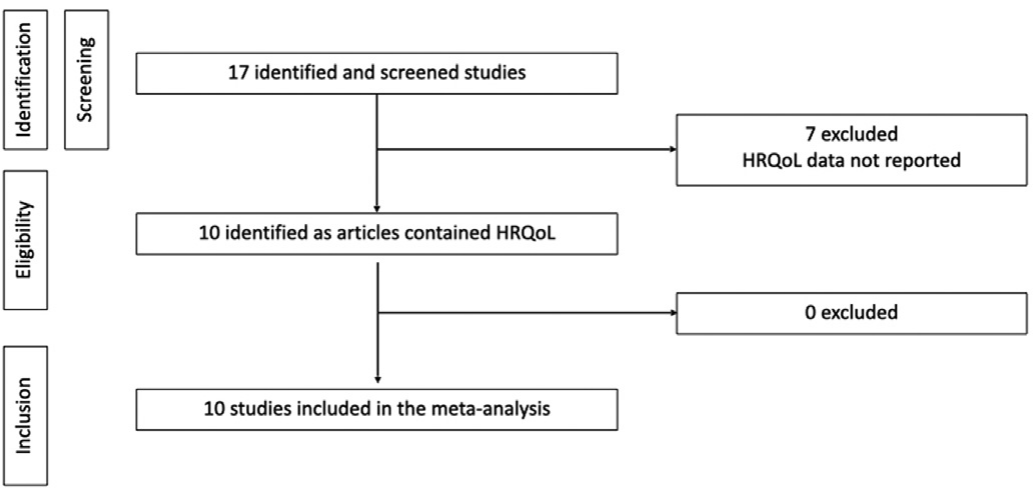
PRISMA flow chart summarizing the process to identify the eligible studies.

We identified all products approved for mBC by the European Medicines Agency based on the European public assessment reports (EPAR) that are publicly available on the agency's website. The following substances, approved by the EMA for the treatment of the HR+/HER2-mBC, have been evaluated: letrozole, anastrozole, exemestane, fulvestrant, ribociclib, palbociclib, abemaciclib, alpelisib [[6], [7], [8], [9], [10], [11], [12], [13], [14], [15], [16], [17], [18], [19], [20], [21], [22], [23], [24], [25]]. The main clinical trials submitted for marketing authorization in the approval procedure were assessed for the presence of the HRQoL in the endpoints, and the corresponding publications concerning the HRQoL analysis were collected. The flow of eligible articles for the meta-analysis was reported following the Preferred Reporting Items for Systematic Reviews and Meta-Analyses (PRISMA) statement (Fig. 1). The trials, with the corresponding and published publication for the HRQoL, were considered for the meta-analysis. All the EPARs available from the EMA website were checked to verify the presence of the HRQoL in the discussion and in the benefit-risk assessment. The related summaries of medicinal product characteristics (SmPCs) were verified to evaluate the presence of the HRQoL data in section 5.1. The time to the publication of the clinical trial and the HRQoL analysis was also taken in account to obtain the mean time of the delay at publication. The results of the HRQoL analysis were collected and a meta-analysis was performed to evaluate the impact of the brand new treatments on HRQoL compared to standard treatments. The main differences between the individual items were also taken into account to highlight any clinically relevant differences and their impact on the risk-benefit discussion.

### 2.1 Statistical analysis

Hazard ratios (HRs) and their corresponding confidence interval (CI) 95% were derived to analyze the global quality of life. Heterogeneity was evaluated by χ2 Q test and I2 statistic [4]. For the Q test, P < 0.05 indicated significant heterogeneity; for the I2 statistic, I2 > 50% was considered significant. The pooled hazard ratio estimate was calculated using a random-effect model [5]. Our results are graphically displayed as forest plots, with hazard ratio < 1.0 indicating better outcome in the experimental arm. Publication bias was evaluated by visual inspection of funnel plots. Calculations were performed using Comprehensive Meta-analysis 3.0 software (CMA; Biostat, Englewood, NJ).

## 3 Results

**Fig. 2:**
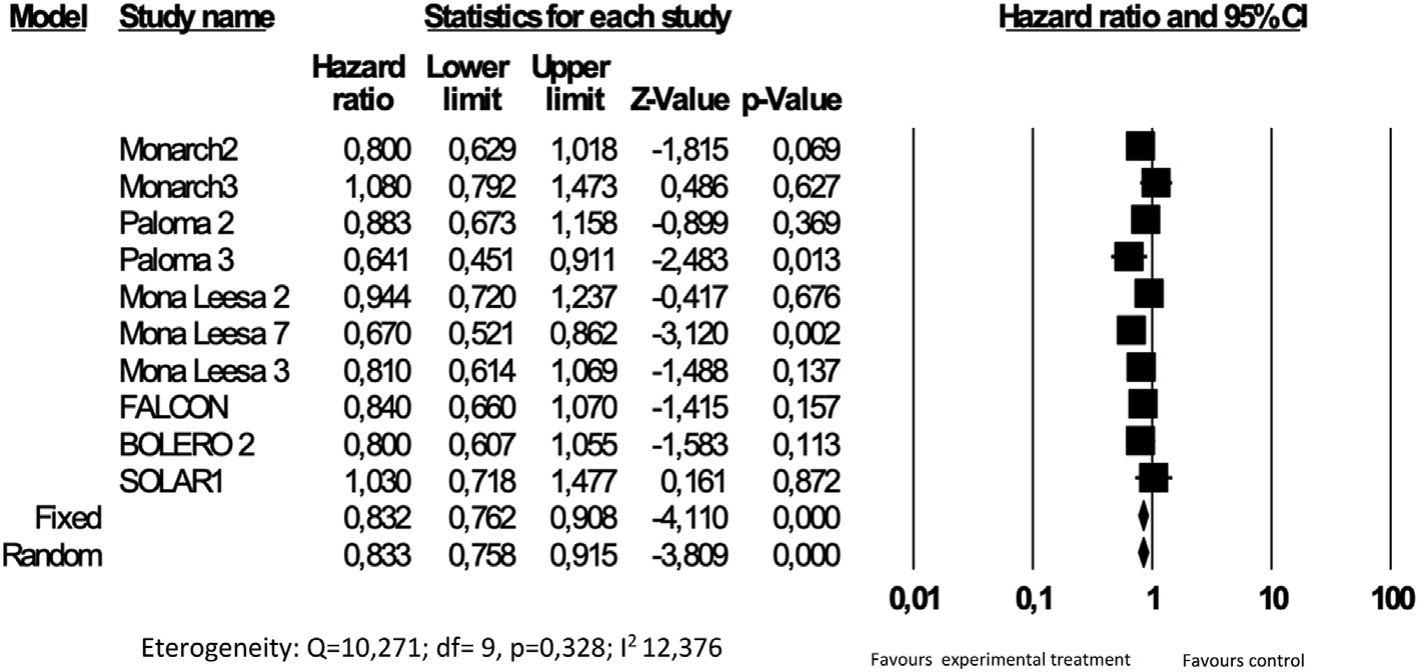
Meta-analysis of the HRs for the GQoL with TTD>10%.

7 out of the 9 active substances taken into account in the current analysis incorporated the HRQoL data in the description of the outcome of the trials. Seventeen trials were identified and in fourteen of these the QoL was included as a secondary endpoint. The time to deterioration (TTD), usually defined as the duration between baseline and first occurrence of a decrease of 5–10 points from basal, with no subsequent increase above these thresholds, represents the more frequently used endpoint across the trials to compare the two treatment arms in term of HRQoL effects. Some key items in the QoL questionnaires used to collect data were also explored as pre-specified analysis. Pain represents the most important item and, more recently, some trials reported the TTD for the pain item score. The results concerning HRQoL were published separately in 10 out of the 17 trials and in the same paper in four of them. The European Organisation for Research and Treatment of Cancer Quality-of-Life questionnaire, Quality of Life Questionnaire Core (EORTC QLQ-C30) questionnaire was used in nine trials. The Functional Assessment of Cancer Therapy for Breast Cancer (FACT-B) was used in 5. The mean time to publication between the clinical results and the QoL data was 1 year (range 0–2). The HRQoL results are reported in terms of improved global quality of life and, only in few cases, a pre-specified analysis is performed for specific scores, mainly the pain score. Overall, only one trial reported a significant improvement of the global QoL (Paloma-3), whereas the remaining trials reported the wording “maintained QoL” or “similar QoL” or “no meaningful differences”. As regards the drug evaluation process from the analysis of the EMA approval documents, the data concerning HRQoL in the EPAR were frequently reported nonextensively. The HRQoL data are contained and discussed in the EPARs of eleven trials considered in the approval process but are cited in only one case (Paloma-3 trial) in the benefit-risk section. In two cases (Monarch-2 and Paloma-3), they are cited in section 5.1 of SmPCs. The meta-analysis was performed on the ten trials for which HRQoL is available. The results showed an overall improvement in global QoL, considering the TTD≥10, (Fig. 2), indicating the consistency of the efficacy of the new substances compared to the standard treatment (Table 1).

**Table 1:** Summary of the quality of life endpoint results and EMA evaluation.

Drug	Authors (study)	QoL Questionnaire	Endpoint	# Pts	HR GQoL (C.I. 95%) [p]	QoL in the SmPC	QoL in the EPAR	QoL cited in the B/R section?	QoL add value to approval?	Results published/simultaneously	Published in the same paper
Abemaciclib	Kaufman [4,5] (Monarch2)	EORTC QLQ-C30, QLQ-BR23	Secondary	669	0,80 (0,63–1,02)	Yes	Yes	No	Yes	Y/N	No
Abemaciclib	Goetz [6] (Monarch3)	EORTC QLQ-C30, QLQ-BR23	Secondary	493	1,08 (0,79–1,47)	Yes	Yes	No	No	Y/N	No
Palbociclib	Rugo [7] (Paloma2)	FACT-B,	Secondary	338	0,883 (0,673–1158	No	No	No	No	Y/N	No
		EQ-5D									
Palbociclib	Harbeck [8] (Paloma3)	EORTC QLQ-C30 v3.0, QLQ-BR23	Secondary	521	0,641 (0,45–0,91)	Yes	Yes	Yes	Yes	Y/N	No
Ribociclib	Verma [9] (MonaLeesa2)	EORTC QLQ-C30v3.0,	Secondary	668	0,59 (0,39–0,87) [p = 0.008]	Yes	Yes	No	No	Y/N	No
		QLQ-BR23, EQ-5D-5L									
Ribociclib	Harbeck [10] (MonaLeesa7)	EORTC QLQ-C30v3.0,	Secondary	116	0,70 (0,53–0,92), [p = 0.004]<	Yes	Yes	No	No	Y/N	No
		QLQ-BR23, EQ-5D-5L									
Ribociclib	Fasching [11] (MonaLeesa3)	EORTC QLQ-C30	Secondary	726	[p < 0.01]	No	No	No	No	Y/N	No
Fulvestrant	Di Leo [12] (CONFIRM)	FACT-B, TOI	Secondary	145	NR	No	Yes	No	No	Y/Y	Yes
Fulvestrant	Osborne [13,15] (9238IL/0021)	FACT-B, TOI	Secondary	851	NR	No	Yes	No	No	Y/Y	Yes
	Howell [14,15] (9238IL/002)										
Fulvestrant	Robertson [16] (FALCON)	FACT-B, TOI	Secondary	145	NR		No	Yes	No	Y/Y	No
Afinitor	Burris [17,18] (Bolero2)	EORTC QLQ-C30v3.0,	Secondary	724	0,74 (0,58–0,95)	Yes	Yes	No	No	Y/N	N
		QLQ-BR23,									
Alpelisib	Mayer [19] (SOLAR1)	EORTC QLQ-C30	Secondary	341	NR	No	Yes	No	No	Y/N	No
Exemestane	Kaufmann [20]	EORTC QLQ-C30	Secondary	769	NR	No	No	No	No	Y/Y	Yes
Letrozole	Mouridsen [21]	/	Not Included	/	NR	/	/	/	/	/	/
Anastrozole	Nabholtz [22]	/	Not Included	/	NR	/	/	/	/	/	/
	Bonneterre [23]										

### 3.1 Analysis of individual items

Looking at the individual items, improvement in pain represents one the main aspects that could have a direct impact on clinicians. Pain is not related to toxicity but is imputable to the burden of disease and its modification is strictly related to the efficacy of a treatment. Eight of the 17 trials reported the results for the pain item. TTD≥ 5–10% in pain is considered to indicate clinical meaningfulness. Regarding the statistical analysis, there was only one trial in which pain was reported as pre-specified for the analysis (Paloma-3 trial). In the Paloma-2 trial, pain was evaluated by the FACTB score item and showed a statistically different decrease in the experimental arm. However, the TTD was not reported. In the Monarch-2 trial, pain was evaluated as a co-primary endpoint combining three different indicators (modified brief inventory short form – mBPI-SF – the pain item from EORTC-QLC30, and analgesic use): although not statistically significant, the TTD of pain appears to be delayed in the experimental arm. In the Monaleesa-3 trial, although no statistical differences were reported in the QLQC30 pain score, the pain severity index was reduced using the BPI-sf. In the Monaleesa-7 trial, although not statistically different, a TTD delay in pain was more consistent within the experimental arm. In the remaining trials, the reduction of pain was reported in the general discussion of the various items and in nine of them no results were presented or discussed (Table 2). Looking at the other items, only a few of them are associated with a trend in improvement for the experimental drug. Some of those results, summarized in Table 3, may be useful for the clinician in weighing the risk-benefit of the treatment proposed.

**Table 2:** Pain item reporting from the pivotal trials.

Drug	Authors (study)	TT(S)D Pain	Results from Pain item QLQC30	Pain item mBPI
Abemaciclib	Kaufman [4,5] (Monarch2)	HR 0,90 (95%CI 0,707–1145) p = 0,40	HR 0,62 (95%CI 0.48–0.79)	HR 0,62 (95%CI 0.47–0.82)
Abemaciclib	Goetz [6] (Monarch3)	/	HR 0,98 (95%CI -2,63–4,58)	/
Palbociclib	Rugo [7] (Paloma2)	/	p = 0,018	/
Palbociclib	Harbeck [8] (Paloma3)	HR 0.642 (95%CI 0.487–0.846) p < 0.001	/	/
Ribociclib	Verma [9] (MonaLeesa2)	/	Mean difference −1952; (95% CI −3826, −79) p = 0.0412	/
Ribociclib	Harbeck [10] (MonaLeesa7)	HR 0.64(95%CI 0.43–0.96)	/	/
Ribociclib	Fasching [11] (MonaLeesa3)	/	HR 1.06 (95%CI 0.74–1.52)	HR 0.77 (95% CI, 0.57–1.05)
Exemestane	Kaufmann [20]	Nr	Exe 51.4% (95% CI, 34.0–68.6)	Nr
			MA 46.2% (95% CI, 30.1–62.8)	

TT(S)D: time to sustained deterioration, HR: Hazard Ratio, MbpimBPI: median brief pain inventory, CI: confidence interval.

**Table 3:** PRO of clinical relevance (results) for the experimental drug derived from specific questionnaires.

Drug	Authors (study)	Item
Abemaciclib	Kaufman [4,5] (Monarch2)	Diarrhea, means difference 24,64 (95% CI 21,58–27,71) p < 0,001
Abemaciclib	Goetz [6] (Monarch3)	Diarrhea HR 1,74 (95% CI 1,25–2,40) p < 0,001
Palbociclib	Rugo [7] (Paloma2)	no other significant items
Palbociclib	Harbeck [8] (Paloma3)	no other significant items
Ribociclib	Verma [9] (MonaLeesa2)	no other significant items
Ribociclib	Harbeck [10] (MonaLeesa7)	Fatigue HR 0,78 (95% IC 0,56–1,1)
Ribociclib	Fasching [11] (MonaLeesa3)	Emotional functioning HR 0,76 (95% CI 0,57–1,01)
		Fatigue HR 0,91 (95% IC 0,68–1,22)
Fulvestrant	Robertson [16] (FALCON)	Functional well-being (p = 0.007)
		Social well-being (p = 0.001)
Exemestane	Kaufmann [20]	Physical functioning, role functioning, global health, fatigue, dyspnea, and constipation (p < 0.01)

CI: confidence interval, PRO: Patient Reported Outcome, HR: Hazard Ratio, CI: Confidence Interval.

### 3.2 Analysis of QoL real world data

In everyday clinical practice, reproducing the improvement in QoL outside the patient selection of a clinical trial is challenging. Few studies are available in literature about the evaluation of QoL in a real-world setting for the substances considered in this analysis. Some real-world studies provide the patients' perspective on pain severity and its impact on health status. In a real-world cross-sectional study, 739 patients were recruited and completed the validated Brief Pain Inventory questionnaire (BPI) for pain severity and the EuroQoL-5D for general health status. A similar proportion of patients (40%) received chemotherapy and endocrine treatment. The first group had a higher rate of moderate/severe level of anxiety/depression compared with patients receiving endocrine therapy in the EQ-5D-3L. Furthermore, pain severity was associated with metastatic site and closely related to health status. In conclusion, the study aimed to demonstrate a better quality of life with endocrine treatment than chemotherapy [26]. Since the recent approval of cyclin-dependent kinase 4/6 inhibitors in combination with endocrine treatment, many real-world reports, while describing patients' characteristics and survival/response outcomes in painstaking detail, only rarely report quality of life data. By way of example, in the Canadian cohort of the Expanded Access Program, PROs were collected only in 97 patients on letrozole plus palbociclib as first-line treatment for mBC, based on the EuroQoL-5D (EQ-5D) questionnaire administered on day 1 of each cycle. The general health status was maintained during treatment with minimal changes from baseline [27]. Among another twenty real-life experiences with palbociclib-based combinations that are available in the literature [28], only in the study by Darden and colleagues was the treatment satisfaction of patients receiving palbociclib evaluated using the Cancer Therapy Satisfaction Questionnaire (CTSQ), with the finding that more than 96% of 604 patients enrolled met or exceeded their expectations regarding the treatment [29]. Even in the mono-institutional case histories of the Modena Cancer Center, no specific endpoints relating to the quality of life of patients are reported, reflecting once again the extreme difficulty in the timely collection and aggregate analysis of these items [30]. In daily clinical practice, reproducing the improvement in QoL outside the patient selection of a clinical trial is challenging. Few studies are available in literature about the evaluation of QoL in a real-world setting for the substances considered in this analysis. Some real-world studies provide the patients' perspective on pain severity and its impact on health status. In a real-world cross-sectional study, 739 patients were recruited and completed the validated Brief Pain Inventory questionnaire (BPI) for pain severity and the EuroQoL-5D for general health status. A similar proportion of patients (40%) received chemotherapy and endocrine treatment. The first group had a higher rate of moderate/severe level of anxiety/depression compared with patients receiving endocrine therapy only in the EQ-5D-3L. Furthermore, pain severity was associated with metastatic site and closely related to health status. In conclusion, the study aimed to demonstrate a better quality of life with endocrine treatment than chemotherapy [24]. The recent approval of cyclin-dependent kinase 4/6 inhibitors (CDK4/6 inhib's) in combination with endocrine treatment in patients has changed the natural history of our patients. Due to the huge clinical impact of these new substances, some trials have been developed and are currently ongoing to evaluate HRQoL in a real-world setting. POLARIS is a prospective, multicenter, non-interventional study that enrolled 1500 patients treated with palbociclib in the USA and in Canada. The study will evaluate the impact on HRQoL, providing real-world data on palbociclib therapy and associated clinical outcomes. RIBANNA is a similar trial, a non-interventional study collecting data on efficacy, safety, duration of therapy and quality of life. Together, these two trials will provide data on the real-world use of CDK4/6 inhibitors. Further experiences are needed for treatments to reduce pain and to preserve health status in patients with HR+/HER2 mBC. ^2^Due to the huge clinical impact of CDK4/6 inhibitors, some trials have been developed and are currently ongoing to evaluate HRQoL in a real-world setting. POLARIS is a prospective, multicenter, non-interventional study aiming to enroll 1500 patients with hormone receptor-positive/HER2-negative mBC treated with palbociclib. The study will evaluate the impact on HRQoL, providing real-world data on palbociclib therapy and associated clinical outcomes [31]. RIBANNA is a similar trial, a non-interventional study collecting data on efficacy, safety, duration of therapy and quality of life: The FACT-B questionnaire will be used to collect PRO data in this trial [32]. Further experiences are needed for treatments to reduce pain and to preserve health status in patients with HR+/HER2 mBC.

## 4 Discussion

The role of HRQoL in the management of cancer patients is sometimes difficult to interpret. Despite the fact that clinical trials experimenting with the newest treatments report quality of life data, although with different times of publication, the importance and impact of these results is scarcely considered. The results from the trials in which QoL data are available are often difficult to compare due to the different questionnaires used and the different ways that the results are reported. Overall, in the trials analyzed in this article, HRQoL is rarely improved in the experimental arm if compared to the standard treatment. This is due to the nature of the instruments used and the presence of multi items/domains involving different aspects of the multi-dimensionality deriving from the QoL. Over the years, the presence of QoL data in the final description of the results has progressively changed, from an increase in the amount of data reported, to a brief description in the final study results paper, and finally a different and separate article. Moreover, the European Society of Medical Oncology (ESMO) has produced a Magnitude of Clinical Benefit Scale (ESMO-MCBS) that includes the QoL [33]. The presence of QoL data in studies is an added value to confirm the advantage of an anticancer treatment if there is an improvement in QoL. An analysis of the EPARs available in the EMA website and repository provided us with the basic data to evaluate the role of the HRQoL information and its impact on the approval decision. Although the HRQoL data are welcomed when a MA holder submits the results of the trial for the approval process, the assessment of QoL is optional. Only a substantial improvement in the results of QoL could be of interest deserving of mention in the assessment report. In some cases the improvement in specific domains that could be of interest for the patient (i.e. pain) are discussed and potentially suitable to be included in section 5.1 of the SmPC. The results of the meta-analysis showed a non-detrimental effect in terms of HRQoL when comparing brand new treatments to the standard treatments. Overall, extracting the Hazard Ratio (HR) of the global QoL and considering TTD≥10, the impact of the active substances considered in this analysis showed a global improvement in QoL (Fig. 2), highlighting the consistency of the efficacy in a clinical context of the most recent agents available for the treatment of mBC. Regarding the analysis of individual items, pain is the most discussed item and the differences in pain score were pointed out in 6 of the 17 trials. Although the results of the pain item may not be comparable, due to the different scales and score used to evaluate it, in all six trials the improvement of the item is highlighted, thus strengthening and corroborating the clinical data derived from the trials. Is this information relevant for clinicians? Does the QoL still have weight in the approval process? We think it is worth having information on QoL improvement in order to allow better communication with the patient by pointing out the relevant information, concerning not only the efficacy and safety in terms of crude numbers, but also the prospect of living a better life during the treatment. The patient aims to be cured but also to maintain wellbeing and a role in society. The clinician should include knowledge of QoL data to prepare the patients in regard to the impact of the treatment on daily life. The interpretation of QoL results is often challenging, but if the clinician pays attention to the results derived from the individual items, useful information may be extrapolated and applied to the practice to allow a better management and more suitable advice for patients. An effort should be made by all the stakeholders to increase the visibility of the HRQoL results in order to allow greater consideration in the assessment during the approval process to make QoL data more easily and visibly available for the clinician and the patients. Measurement of quality of life in the real world is lacking in results due to low interest in evaluating this field in the clinical context. This represents an unmet need that could be of interest in the evaluation of a conditional marketing authorization. The international agencies have produced specific documents in which the role of HRQoL has been discussed. The Food and Drug Administration (FDA) released guidance for the company to support labeling claims, whereas the European Medicine Agency (EMA) released a reflection paper in which the place of HRQoL in the drug evaluation process is discussed [34,35]. An increasing number of new targeted oral substances have been released in the last twenty years for the treatment of endocrine sensitive breast cancer. For these treatments, which include hormone-therapy substances, m-TOR inhibitors and the brand new CDK 4/6 and P3IK inhibitors, QoL aspects assume an important role. The targeted substances have a different safety profile to classic hormone-therapy. The increased incidence of metabolic adverse events (AEs) and their peculiar toxicities (i.e. non-infectious pneumonitis) lead the physician to increase the attention paid to quality of life aspects before choosing a given therapy. Data from real-world studies could help to address the impact in the unselected population of the brand new substances in clinical practice but no studies are available to evaluate the impact in practice. The evolution of QoL measurement has also led to the development of a new tool to evaluate the patient's symptoms. The PRO-CTCAE tool has been developed and validated [36]. The purpose of PRO-CTCAE is to improve the evaluation of toxicity by focusing on the patient's point of view using a simple tool containing some questions that are not included in the classic HRQoL instrument. The true value of PROs collected through HRQoL questionnaires rather than common toxicity criteria, in order to obtain the patient's perspective on toxicity reporting (PRO-CTCAE), is still a matter of debate. Their use is gradually expanding and their role could be helpful in the future evaluation of new substances, improving the quality of side-effect data collection in clinical trials [37]. It is also worth having any additional information on PROs in the post period for progression to reinforce the impact of the substances used in the first instance and which could help to describe a possible continuum in maintaining QoL that, with treatment sequences, is difficult to perceive. For these reasons it may be worth including the HRQoL results derived from the trial in the dossier submitted for a marketing authorization and to evaluate in greater depth the quality of life data in the EPAR. The evaluation should be also reflected in the summary of medicine product characteristics (SmPC) in order to increase the amount of information provided to the physician.

## Funding

This research did not receive any specific grant from funding agencies in the public, commercial, or not-for-profit sectors.

FACT-B: Functional Assessment of Cancer Therapy for Breast Cancer, EORTC QLQ-C30: European Organisation for Research and Treatment of Cancer Quality-of-Life questionnaire, Quality of Life Questionnaire Core, QLQ-BR23: Quality of Life Questionnaire Core Breast Cancer, TOI: Trial Outcome Index, EQ-5D: Euro Quality Of Life 5 dimensions, TT(S)D: time to sustained deterioration, HR: Hazard Ratio, mBPI: median brief pain inventory, CI: confidence interval.
